# CFS in Children and Adolescent: Ten Years of Retrospective Clinical Evaluation

**DOI:** 10.1155/2013/270373

**Published:** 2013-06-16

**Authors:** Irene Elgen, Omar Hikmat, Tora N. Aspevik, Ellen Merete Hagen

**Affiliations:** ^1^Department of Child and Adolescent Psychiatry, Haukeland University Hospital, 5021 Bergen, Norway; ^2^Department of Clinical Medicine, University of Bergen, P.O Box 7804, 5020 Bergen, Norway; ^3^Department of Paediatrics, Haukeland University Hospital, Jonas Lies Vei 65, 5021 Bergen, Norway

## Abstract

*Aim*. To estimate number of children being diagnosed with chronic fatigue syndrome (CFS). *Methods*. For a period of 10 years (2002–2011) data from children being referred for fatigue symptoms were collected retrospectively. *Results*. Thirty-seven children were referred. Four were excluded due to incorrect coding. Six (18%) patients received other diagnoses at the end of evaluation time. Of the 27 who received the diagnosis G93.3, four had a previous chronic illness, while 23 patients were previously healthy. All patients reported onset of fatigue symptom in relation to an infection, and all tested positive for IgG to either Epstein-Barr virus, cytomegalovirus or borrelia, indicating previous infection. There were 16 (59%) boys among the 27 patients. The mean age at the debut of fatigue symptoms was 141 months (SD 30) for boys and 136 months (SD 31) for girls, respectively. Being underweight, defined as BMI < 17.5, was found in 12 (44%) patients. *Conclusion*. An increasing number of children and adolescents are evaluated for CFS. The clinical assessment of children and adolescents with possible CFS need systematically evaluation. Nutritional status, possible eating disorder, and psychosocial issues need to be addressed and evaluated carefully. A multidisciplinary approach is essential when assessing CFS in children and adolescents. There is a need for European guidelines.

## 1. Introduction

During the last years an increased focus on fatigue symptoms/syndrome among children has been observed, together with an increase in referrals to our hospital. Children with fatigue have always been evaluated in primary care. Whether these illnesses are increasing in number or just an increase in referrals to the specialist, in part based on greater interest in the topic is unknown.

The aetiology of the condition is still unclear after almost three decades of research in this area, where infectious, immunological, neuroendocrine, sleep, and psychiatric mechanisms, among others, have been investigated [1]. Despite the fact that no evidence has been found so far of an infectious aetiology, it has been proposed that a heterogeneous group of infections may trigger or maintain the symptoms of CFS [1]. Abnormalities in the immune system have been reported in CFS-patients, among these, deficiencies in natural killer cell function and increased expression of activation markers on the cell-surface of T-lymphocytes, especially increased numbers of CD8+ cytotoxic T cells that have certain antigenic markers [2]. Other studies have described higher frequencies of autoantibodies. Whether these abnormalities have any relationship to CFS and its symptoms remains unclear [3]. 

The prevalence of the condition among young adults in the twenties and older has been reported as three percent [4]. Few have studied the prevalence of CFS in children and adolescence [5–7]. Studies have found a higher prevalence of CFS of two to three among females [1, 4, 8, 9]. Studies have reported successful treatment trials with cognitive therapy [10, 11]. Krilov et al. found that children and adolescents with chronic fatigue have a syndrome that is similar to that described in adults but that the syndrome presents earlier in the course of the illness and has a more optimistic outcome [12]. A doctoral thesis from Norway did not find socioeconomic status to contribute to the fatigue symptom [13], opposite to what Chalder et al. found [14].

Having a disabling condition like CFS in childhood and adolescence may pose major consequences regarding mental health, personal relationships, school attendance and participating in social life and work. This may also apply to the whole family of a child suffering from CFS. It is important to ensure early and proper management of these patients [15, 16]. 

The aim of the present hospital-based clinical retrospective study was (1) to estimate the number of children being diagnosed with G93.3 postviral fatigue syndrome during a 10-year period (2002 to 2011) and (2) describe the clinical and laboratory findings in children with postviral fatigue syndrome.

## 2. Method

### 2.1. G93.3 Postviral Fatigue Syndrome

The diagnosis given in ICD-10 G93.3 Postviral fatigue syndrome (CFS) is also used diagnosing children and adolescent with fatigue [17]. However, time aspect with onset of symptoms is shorter for children than adults. Three month is recommended in the Norwegian guidelines for CFS. All children and adolescents in the county where this study took place, was referred to specialist when diagnosing G93.3 post viral fatigue syndrome. All children suffering from fatigue are investigated in the Department of Paediatrics. During the first consultation the specialist will apply a preliminary diagnosis while the medical investigations are done. At the end of evaluation, the final diagnosis is entered in the patient record. It should be noted that not all children with illness compatible with CFS will also fulfil criteria for postviral fatigue syndrome.

### 2.2. Subjects

The study was retrospective, and the subjects were all children under the age of 16. A list of patients who underwent investigations and were diagnosed with G93.3 postviral fatigue syndrome according to the ICD-10 classification was collected from the patient registry. The diagnosis was set by a paediatrician either during or at the end of the evaluation period. The data were collected during the period 2002 to 2011.

### 2.3. Medical Record

From the medical record the following data were collected: (1) clinical examination, (2) blood sample tests, (3) other investigations, and (4) evaluation by physiotherapist, psychiatrist evaluating depression and anxiety using ICD-10 criteria [17], and neuropsychologist assessing WISC-R measuring IQ [18]. Body mass index (BMI) was calculated retrospectively based on weight and length as kg/m^2^. We defined overweight as BMI > 25 and underweight as BMI < 17.5 [8]. Because some findings support the notion that CFS may be due to one or more immune disorders that have resulted from exposure to an infectious agent [19], IgG antibodies to Epstein-Barr virus (EBV) and cytomegalovirus (CMV) were used as a measure of previous infection.

## 3. Results

**Figure 1:**
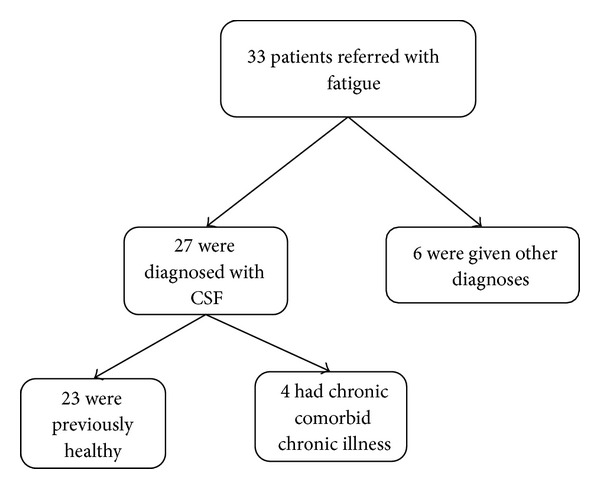
Number of children referred for CFS/ME over a period of 10 years (2002–2011).

A total number of 37 patients were referred with fatigue symptoms according to the patient registry. Four patients were excluded due to obvious incorrect coding. Of the remaining 33, six (18%) patients received other diagnosis at the end of evaluation time. All patients were referred within the last three years. Two were diagnosed with epilepsy, two with sleeping disorders, one with hypothyreosis and one did not fulfil the criteria of CFS or any other diagnosis. Of the 27 who received the diagnosis G93.3, four had comorbid chronic illness. The remaining 23 patients were all previously healthy (Figure 1). All 27 patients reported onset of fatigue symptom in relation to an infection. 

**Figure 2:**
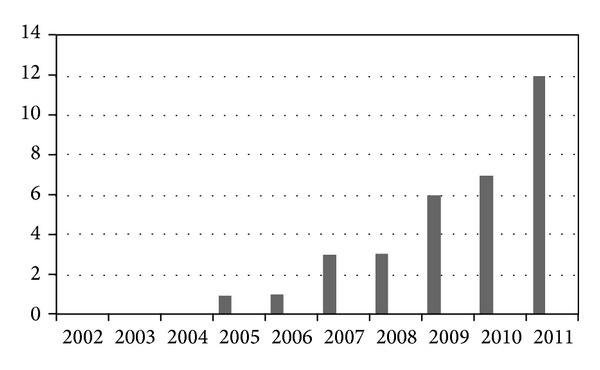
Number of children referred for fatigue symptoms (2002–2011).

There were an increased number of referred patients with fatigue during the 10-year period, with almost three in four patients investigated during the last three years (2009–2011). The annual referral is given in Figure 2. Previous studies have found that many children with CFS do have mental health problems irrespective of cause [20–23]. According to the psychiatrist assessment, none of the patients suffered from anxiety or depression. No cognitive delay (IQ < 70) was found. 

The referred fatigue patients underwent additional medical investigations (Table 1). Three children had a pathological EEG; two of them had epilepsy and one sleeping disorder. Heart rate (HR) and blood pressure (BP) were all within normal range.

Additional blood tests and laboratory tests are described in Table 2. Three patients had increased cobalamin and one increased TSH/FT4 ratio. All tests were normal on followup. One child had low FT4 and later diagnosed as hypothyreosis. 

Among the 27 patients referred with CFS, 16 (59%) were boys. The mean average age for the onset of fatigue symptoms was 141 months (SD 30) for boys and 136 months (SD 31) for girls, respectively. Being underweight, defined as BMI < 17.5, was found in 12 (44%), while one (4%) was overweight with a BMI > 25. Half of the underweight children were boys.

Of the 27 patients with CFS (G93.3), 20 (74%) tested positive for IgG to Epstein-Barr virus, six (22%) tested positive for IgG to cytomegalovirus, and one (4%) tested positive to borrelia, indicating previous infection.

## 4. Discussion 

In the present clinical retrospective study we found no gender differences among children and adolescent younger than 16 years of age with chronic fatigue symptom, with debut of symptoms after 10 years of age. Almost half of them were underweight and half of these were boys. Almost all patients reported a previous infection as the onset of fatigue symptom. Almost one in five suffering from fatigue symptoms proved to have other diagnoses.

During the last decade, there have been an increase in number of referrals of children with fatigue as the only symptom. The reason for these increased numbers is uncertain. Chronic fatigue syndrome is not a new syndrome, there are documented cases since the 19th century [24]. However during the last decade, there has been an increased knowledge and awareness about the condition in the healthcare system and in the media in Norway which influence the number of referrals. 

In this study, the use of medical and biochemical test when assessing children and adolescence with chronic fatigue syndrome varied. The diagnosis was changed in almost one in five patients. The increased focus on the CFS diagnosis may have led to excessive use during the diagnostic evaluation process. 

The strength of the study is the retrospective design which allows us to confirm the diagnoses. The weakness is the low number of children included in the study. In addition, due to uncertainty whether the whole county was covered, one cannot estimate incidences. 

Previous studies have reported a higher prevalence of CFS in the female population [25, 26]. We did not find a gender difference. However, this was a very small population, and it is possible that the results could have been different in a larger group. Time of onset seemed to be in accordance with other studies who describe symptom onset of fatigue between almost twelve until fifteen years [26]. 

Almost all patients reported themselves to be previously healthy prior to their fatigue and also ascribed the onset due to an infection. One in six had a chronic condition that could explain their fatigue symptoms but still fulfilling criteria for G93.3 postviral fatigue syndrome. This is in accordance to previous publications [4, 26]. The diagnosis of CFS is usually established after excluding other chronic conditions; however, long standing medical or psychological conditions can also result in CFS. There is a gray area between fatigue due chronic medical and psychological conditions and fatigue which is a part of CFS. This zone needs to be examined carefully, and diagnostic criteria for CFS should be adjusted to clarify this overlap. 

Interestingly, we found that more than one in three of the referred patients was underweight, with an equal gender representation. The question is whether the underweight as a cause of fatigue has been overlooked in the diagnostic process or the CFS itself is the reason for underweight. Lack of the BMI records before the onset of fatigue symptoms makes it difficult to answer this question in our study. This is opposite to a recent study where high BMI was found to be significantly associated with prolonged duration of CFS in a retrospective cohort of children [27]. Many of the adolescents had additional symptoms from the gastrointestinal system, like diarrhea and nausea [12, 28]. This may possibly partly explain the low BMI. Another possible reason could be the loss of muscle mass due to inactivity. Further studies are needed to address the issue of eating disorders and to explain the reason for the low BMI. Many medical conditions present with long lasting fatigue as a disabling symptom [29, 30]. In this retrospective study, one in five referred with fatigue symptom received other diagnoses. These diagnoses were mainly epilepsy, sleeping disorders and hypothyreosis. This is a reminder of the importance of a thorough investigation of the children being evaluated for possible CFS, to exclude other causes for their fatigue. 

## 5. Conclusion

An increasing number of children and adolescents are evaluated for CFS. The clinical assessment of children and adolescents with possible CFS needs systematic investigation. Nutritional status, possible eating disorder, and psychosocial issues need to be addressed and evaluated carefully. A multidisciplinary approach is essential when assessing CFS in children and adolescents. There is a need for European guidelines.

## Figures and Tables

**Table 1:** Medical examination in a population of children and adolescents with fatigue symptoms (*N* = 33).

Examinations performed as part of CFS evaluation (*N* = 33)		
Number of patients being examined and number of pathologic findings		
	Numbers (%)	Pathology (%)
MR cerebrum	29 (87.8)	0 (0.0)
Chest X-ray	18 (54.5)	0 (0.0)
Abdominal ultrasound	20 (60.5)	0 (0.0)
EEG	27 (81.7)	3 (9.0)
ECG	20 (60.6)	0 (0.0)

**Table 2:** Biochemical tests in a population of CFS children and adolescents.

Number of patients tested and pathological findings (*N* = 33)		
	Number (%)	Pathology (%)
Gluten antibodies	21 (63.6)	0 (0.0)
Anti-nucleus antibodies	14 (42.4)	0 (0.0)
Cobalamin	23 (69.7)	3 (9.1)
Folate	23 (69.7)	0 (0.0)
TSH/FT4	30 (91.0)	2 (15.2)
Cortisol	10 (30.3)	1 (3.0)
ACTH	5 (15.2)	0 (0.0)
CK	24 (72.7)	1 (3.0)
ASAT	24 (72.7)	0 (0.0)
Creatine	31 (93.9)	0 (0.0)
CRP	30 (90.9)	0 (0.0)
Haemoglobin	33 (100)	1 (3.0)
SR	29 (87.8)	1 (3.0)
Urine dip sticks	19 (57.5)	0 (0.0)
